# Evaluation of Kilifi Epilepsy Education Programme: A randomized controlled trial

**DOI:** 10.1111/epi.12498

**Published:** 2014-01-21

**Authors:** Fredrick Ibinda, Caroline K Mbuba, Symon M Kariuki, Eddie Chengo, Anthony K Ngugi, Rachael Odhiambo, Brett Lowe, Greg Fegan, Julie A Carter, Charles R Newton

**Affiliations:** * KEMRI-Wellcome Trust Research Programme, Centre for Geographic Medicine Research (Coast)Kilifi, Kenya; † Nuffield Department of Clinical Medicine, Centre for Clinical Vaccinology and Tropical Medicine, University of OxfordOxford, U.K; ‡ Department of Psychiatry, University of OxfordOxford, U.K; § Research Support Unit, Faculty of Health Sciences, Aga Khan University (East Africa)Nairobi, Kenya; ¶ Institute of Child Health, Centre for International Health and Development, University College LondonLondon, U.K; # Neurosciences Unit, Institute of Child Health, University College LondonLondon, U.K; ** Clinical Research Unit, London School of Hygiene and Tropical MedicineLondon, U.K

**Keywords:** Epilepsy, Education intervention, Adherence, Beliefs about epilepsy, Seizure frequency

## Abstract

**Objectives:**

The epilepsy treatment gap is largest in resource-poor countries. We evaluated the efficacy of a 1-day health education program in a rural area of Kenya. The primary outcome was adherence to antiepileptic drugs (AEDs) as measured by drug levels in the blood, and the secondary outcomes were seizure frequency and Kilifi Epilepsy Beliefs and Attitudes Scores (KEBAS).

**Methods:**

Seven hundred thirty-eight people with epilepsy (PWE) and their designated supporter were randomized to either the intervention (education) or nonintervention group. Data were collected at baseline and 1 year after the education intervention was administered to the intervention group. There were 581 PWE assessed at both time points. At the end of the study, 105 PWE from the intervention group and 86 from the nonintervention group gave blood samples, which were assayed for the most commonly used AEDs (phenobarbital, phenytoin, and carbamazepine). The proportions of PWE with detectable AED levels were determined using a standard blood assay method. The laboratory technicians conducting the assays were blinded to the randomization. Secondary outcomes were evaluated using questionnaires administered by trained field staff. Modified Poisson regression was used to investigate the factors associated with improved adherence (transition from nonoptimal AED level in blood at baseline to optimal levels at follow-up), reduced seizures, and improved KEBAS, which was done as a post hoc analysis. This trial is registered in ISRCTN register under ISRCTN35680481.

**Results:**

There was no significant difference in adherence to AEDs based on detectable drug levels (odds ratio [OR] 1.46, 95% confidence interval [95% CI] 0.74–2.90, p = 0.28) or by self-reports (OR 1.00, 95% CI 0.71–1.40, p = 1.00) between the intervention and nonintervention group. The intervention group had significantly fewer beliefs about traditional causes of epilepsy, cultural treatment, and negative stereotypes than the nonintervention group. There was no difference in seizure frequency. A comparison of the baseline and follow-up data showed a significant increase in adherence—intervention group (36–81% [p < 0.001]) and nonintervention group (38–74% [p < 0.001])—using detectable blood levels. The number of patients with less frequent seizures (≤3 seizures in the last 3 months) increased in the intervention group (62–80% [p = 0.002]) and in the nonintervention group (67–75% [p = 0.04]). Improved therapeutic adherence (observed in both groups combined) was positively associated with positive change in beliefs about risks of epilepsy (relative risk [RR] 2.00, 95% CI 1.03–3.95) and having nontraditional religious beliefs (RR 2.01, 95% CI 1.01–3.99). Reduced seizure frequency was associated with improved adherence (RR 1.72, 95% CI 1.19–2.47). Positive changes in KEBAS were associated with having tertiary education as compared to none (RR 1.09, 95% CI 1.05–1.14).

**Significance:**

Health education improves knowledge about epilepsy, but once only contact does not improve adherence. However, sustained education may improve adherence in future studies.

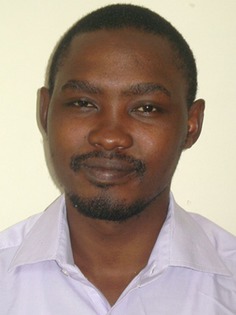



**Fredrick Ibinda** is a statistician at KEMRI-Wellcome Trust Research Programme in Kilifi, Kenya.

Epilepsy is most prevalent in low income countries (LICs).^1^ can be managed successfully, with >70% of people with epilepsy (PWE) achieving full seizure control or significant reduction in seizure frequency following effective use of antiepileptic drugs (AEDs).^2^ However, a significant number of people with active epilepsy still do not receive appropriate treatment, what is referred to as “the epilepsy treatment gap” (ETG).^3^ In systematic reviews, the ETG ranged from 31 to 100% in low and middle income countries, with the highest ETGs being in rural areas and LICs.^2^,^4^ The gap is influenced by limited knowledge of epilepsy, cultural beliefs, untrained health workers, cost of treatment, and unavailability of AEDs.^2^ Recently we showed that cultural beliefs and lack of knowledge about epilepsy are important risk factors for ETG and that adherence may be improved by at least 20%, if beliefs could be modified.^5^ Most PWE in LICs may only access specialist services once in their lives.

Previous studies have suggested that health education may encourage AEDs use.^6^,^7^ A randomized controlled trial of the Modular Service Package Epilepsy (MOSES) in Europe found that patient education improved knowledge about epilepsy, coping strategies, and seizure outcome, but this study did not investigate improvement in adherence.^8^ Demonstration projects in rural China improved biomedical care using education and treatment interventions,^9^,^10^ but were not tested with randomized controlled trials. The findings from these two studies cannot be extrapolated to Africa due to different sociocultural backgrounds and health systems.

We assessed the impact of a one-day low-cost educational intervention on adherence to AEDs, seizure frequency, and Kilifi Epilepsy Beliefs and Attitudes Scores (KEBAS).

## Methods

### Study setting

The study was conducted in the Kilifi Health and Demographic Surveillance System (KHDSS), in which there were 261,919 residents in 2011.^11^ Most people are Giriama (45%), and about 55% of the population is considered poor; 80% depend on subsistence farming. KHDSS is served by one district level hospital, Kilifi District Hospital (KDH), which stocks four AEDs: phenobarbital, phenytoin, carbamazepine, and sodium valproate. There are 13 health clinics and dispensaries that stock only phenobarbital, although the supply is erratic.

### Study participants

This study is part of an epidemiologic survey of epilepsy conducted in 2008, in which 738 people of all ages had active convulsive epilepsy, defined as at least two unprovoked convulsions, with one in the 12 months prior to being assessed.^12^ Recruitment of PWE involved in this study started in August 2009.

### Randomization and masking

**Figure 1:**
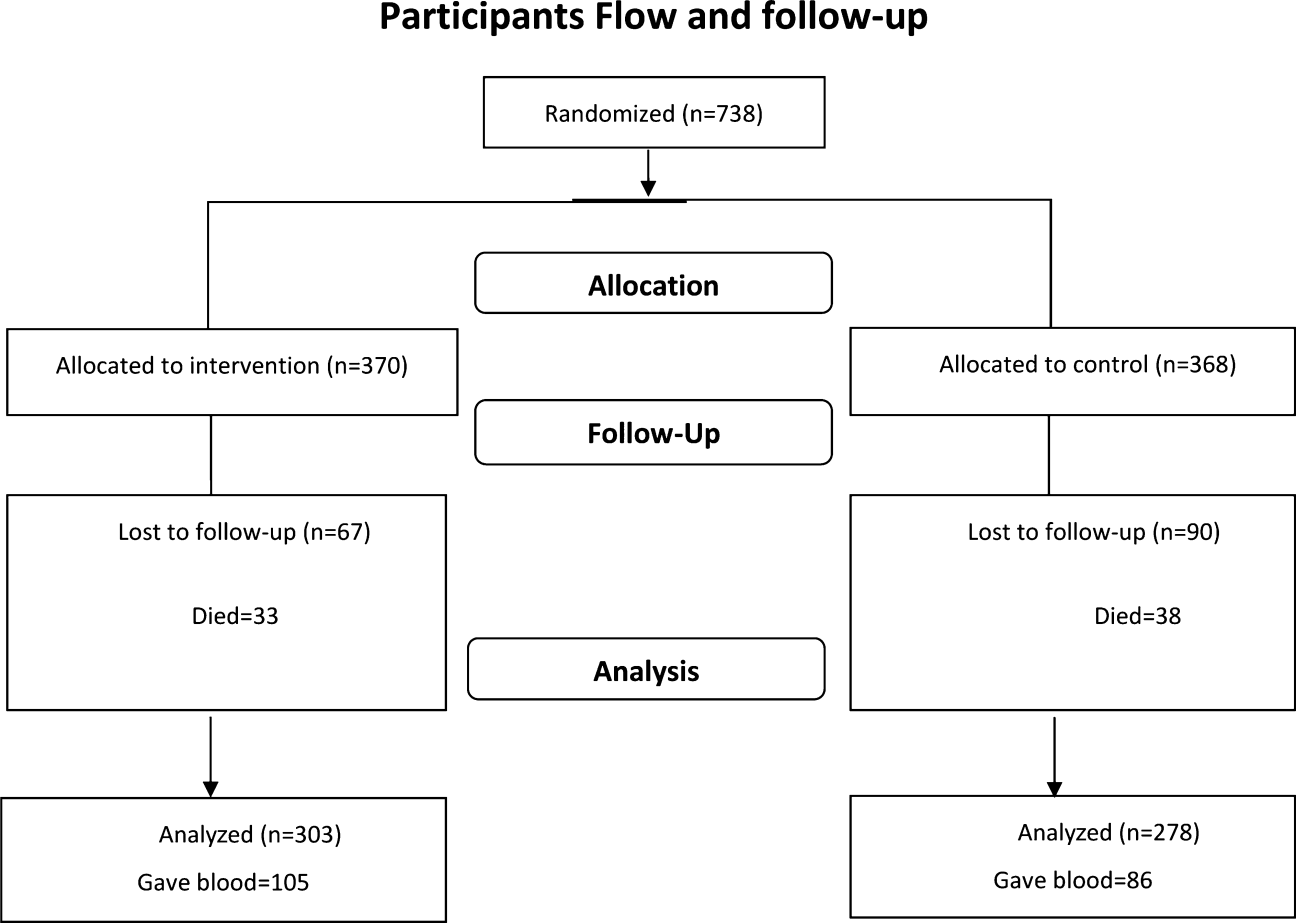
A flow chart representation of the participant flow. Seven hundred thirty-eight people with epilepsy (PWE) were randomized, but analysis was done for the 581 who were observed at both the beginning and end of the study. Assays of antiepileptic drugs were done on 105 in the intervention and 86 in the nonintervention group who provided blood samples.

The data manager used computer-generated randomization to allocate the 738 participants to either the intervention or nonintervention group (Fig.1). The laboratory technicians conducting the assays were blinded to the randomization. The questionnaires were administered by trained field staff, both at baseline and follow-up.

### Study design

The participants completed questionnaires immediately before the educational intervention and 1 year after the education intervention was implemented in the intervention group (Fig. S1). The nonintervention group received the health education after the second assessment. If the PWE was a child or was cognitively impaired, the questionnaire was administered to a caregiver.

### Educational intervention

The educational intervention was only randomized in the PWE and caregivers. In addition, discussions with traditional healers and sensitization of medical providers occurred. The intervention was designed and delivered by a team of epilepsy researchers and field staff who had a good relationship with the community.

#### Educational intervention for PWE and caregivers

The PWE and an identified designated supporter (caregiver) were invited to a one-day education program on epilepsy, types of seizures, causes of epilepsy, effects of epilepsy on child development, treatment of epilepsy, side effects of drugs, drug safety, what to do during a seizure, when to take a PWE to hospital, prevention of epilepsy, what PWE can and cannot do, and advice to families. The intervention was tested as one contact, since most patients in Africa may only be seen once a year at a center with specialists. Only one workshop was held per week. The intervention took 5 months to deliver. The workshop consisted of a maximum of 20 people, and a total of 19 workshops were conducted. Techniques such as role-plays, picture materials, group discussions, songs, and narratives from PWE were used. The training was delivered in the participants' native language (Kigiriama) and Kiswahili. A brochure written in Kiswahili detailing all the topics discussed was given to each participant. The workshops were scheduled with a clinic visit.

#### Discussions with traditional healers

Although the original design was to randomize the traditional healers consulted by the PWE, most traditional healers could neither be traced nor matched to PWE. Consequently, 51 traditional healers were invited to a one-day workshop in which the topics were similar to those discussed with the PWE.

#### Sensitization of medical providers

Fourteen health providers (nurses and clinical officers) from public health facilities within KHDSS attended a one-day workshop on epilepsy management. Topics discussed included epidemiology of epilepsy, definition of epilepsy, seizures and other terminologies, causes of epilepsy, common precipitating factors of epilepsy, international classification of epileptic seizures, diagnosis of epilepsy, differential diagnosis of epilepsy, conditions coexisting with epilepsy, management of epilepsy, and clinical clerking skills.

### Outcomes

The primary outcome was improvement in adherence of PWE to AEDs as measured by self-reports and drug levels in the blood. Secondary outcomes were comparison of seizure frequency, and KEBAS between the intervention and nonintervention group. These primary and secondary measures were also compared between the baseline and end of the study. Seizures were defined as less frequent if the patients experienced ≤3 seizures in the last 3 months. In this study, “reduced seizures” was defined as a reduction in seizure frequency at the end of the study compared to baseline.

#### Measuring blood level adherence

Blood samples were assayed for the most commonly used AEDs (phenobarbital, phenytoin, and carbamazepine). Plasma drug concentrations were measured using a fluorescence polarization immunoassay analyser (TDxFLx Abbott Laboratories, Abbott Park, IL, U.S.A.). The detectable ranges for the different drugs were the following: phenobarbital 1.1 μg/mL, phenytoin 1.0 μg/mL, and carbamazepine 0.5 μg/mL.^13^ The optimal ranges were defined as follows: phenobarbital 10–40 μg/mL, phenytoin 10–20 μg/mL, and carbamazepine 4.0–12 μg/mL.^14^ An individual was defined as adherent if AEDs were detectable in their blood, and improved adherence if the AED levels were within the optimal levels at follow-up. There were no obvious serious risks in this study except for slight pain due to needle pricks for which the PWE were appropriately counseled.

#### Questionnaires

The questionnaires for the study were developed in English, translated into the local language, and then back-translated. Questions were grouped into four categories: sociodemographic characteristics (age, sex, religion, and education), severity of epilepsy (frequency of seizures, injury during a seizure, and number of medications [monotherapy or polytherapy]); adherence (details of taking prescribed medication regimen); and questions on epilepsy beliefs and attitudes.

PWE were asked questions regarding the AEDs they were currently taking and in addition requested to display them on a board to aid recognition. Self-reported adherence was assessed using a four-item Morisky medication adherence scale that has been used in other epilepsy studies.^5^,^15^,^16^

Epilepsy beliefs and attitudes were measured using KEBAS,^17^ which has 34 questions that constitute five subscales (causes of epilepsy, biomedical treatment of epilepsy, cultural treatment of epilepsy, risk and safety concerns, and negative stereotypes about epilepsy). Higher scores reflected more positive beliefs and attitudes about epilepsy.

### Ethical considerations

Written informed consent was obtained from all study participants or guardians. Approval for the study was obtained from the Kenya Medical Research Institute National Ethical Review Committee. This study is registered in ISRCTN register under ISRCTN35680481 and follows the consort guidelines.^18^ The full protocol can be accessed from the KEMRI/Wellcome trust website http://www.kemri-wellcome.org/projects/406.

### Statistical analysis

An estimated sample size of 600 PWE with equal numbers in each group provided 99% power to detect a 20% change in adherence to AEDs from 30% to 50%. Data were double entered and verified in MySQL. All statistical analyses were performed using STATA version 12 (STATA Corp, College Station, TX, U.S.A.). We used Pearson's chi-square to compare the proportion of PWE adhering to AEDs and with less frequent seizures between intervention and nonintervention groups at the end of the study, and between baseline and follow-up. In addition, logistic regression was also used to compare the odds of adhering to AEDs at the end of study between the intervention and nonintervention groups. KEBAS between the groups were compared using *t*-tests. Other quantitative variables were compared using *t*-test, whereas Pearson chi-square was used for categorical variables.

Following the improvement in adherence, in which 29 of 83 PWE transit from nonoptimal AED levels at baseline to optimal levels at follow-up, we did a post hoc analysis of the factors that could be associated with that improvement. Similarly, this was done for 347 PWE who had changes in seizure frequency, of whom 244 had a reduction in seizures, and for changes in KEBAS where 534 of 581 had a positive change in scores. In this analysis, improvement in KEBAS was defined as an improvement in scores in at least one of the items of KEBAS. Demographic factors included in the analyses were age, sex, religious affiliation, and education level. On the other hand, epilepsy-related variables included learning difficulties, neurologic deficits, number of medications (monotherapy or polytherapy), and whether one had injuries. Modified Poisson regression was used to ascertain relative risks, which unlike the odds ratios do not overestimate the effect size when the rare event assumption is violated.^19^ Relative risks are preferred over odd ratios in most prospective studies.^20^,^21^ These analyses were adjusted for the intervention because they were done for the two groups combined. We first examined univariate associations and the variables with p-values < 0.25 were retained in the final multivariable models to identify the independent associations. p-Values < 0.05 were considered statistically significant.

## Results

### Study participants

Data were analyzed for 581 PWE observed at both time points. At baseline, the two groups had similar social demographic and clinical characteristics (Table1). In the 157 PWE not seen after 1 year (because of death [45.2%], outmigration [48.4%], and withdrawal [5.7%] [Fig.1]), there were no statistically significant differences in demographic and epilepsy-related characteristics between the groups. A significantly higher proportion of those not seen after one year had more cognitive impairment, learning difficulties, and were on polytherapy compared to the 581 who were seen.

**Table 1:** Baseline demographic and clinical characteristics

Variable	Intervention group (N = 303)		Nonintervention group (N = 278)		p-Value
	n	%	n	%	
Age: Mean (SD)	303	19.2 (17.4)	278	19.5 (15.6)	0.86
Female	143	47.2	138	49.6	0.56
Religion					
Traditional	127	41.9	128	46.0	
Christian	136	44.9	122	43.9	
Islam	40	13.2	28	10.1	0.41
Education level					
None	142	46.9	114	41.0	
Primary	138	45.5	142	51.1	
Secondary	17	5.6	20	7.1	
Tertiary	6	2.0	2	0.7	0.23
Learning difficulties	96	31.9	85	30.6	0.77
Neurologic deficits	70	23.1	54	19.4	0.28
On polytherapy	54/154	35.1	57/154	37·0	0.72
Seizure frequency (last 3 months)					
None	96	31.9	91	32.7	
1–3	91	30.0	95	34.2	
4–6	45	14.9	34	12.2	
>6	71	23.4	58	20.9	0.58
Adherence					
Self-reported	52/195	26.7	54/199	27.1	
Blood levels, detectable	71/195	36.4	76/199	38.2	
Blood levels, optimal	52192	27.1	59/196	30.1	

KEBAS	n	Mean (SD)	n	Mean (SD)	p-Value
Beliefs about causes of epilepsy	303	7.3 (2.8)	278	7.3 (2.9)	0.82
Beliefs about biomedical treatment	303	15.0 (2.0)	278	14.9 (2.2)	0.63
Beliefs about cultural treatment	303	11.0 (4.8)	278	11.1 (4.9)	0.79
Beliefs about risks of epilepsy	303	7.4 (1.3)	278	7.5 (1.2)	0.41
Stereotypes about epilepsy	303	8.4 (4.2)	278	8.5 (4.6)	0.72

If all the data were not available, both the numerators and denominators are provided.

At the end of the study, only 105 PWE from the intervention group and 86 from the nonintervention groups gave blood samples. In both groups combined, phenobarbital was detected in 84, phenytoin in 58, and carbamazepine in 75. Ninety-three people (48.7%) had optimal drug levels, 62 on phenobarbital, 3 on phenytoin, 24 on carbamazepine, and 2 on both phenobarbital and carbamazepine. In comparison to those that gave blood samples, those who did not give samples held significantly more traditional religious and cultural beliefs, and believed that AEDs caused epilepsy. The self-reported adherence had low sensitivity (46.2%, 95% CI 35.8–56.9%) and specificity (40.8%, 31–51.2%) compared to adherence based on optimal levels. Similarly the sensitivity (46.3%, 38.1–54.7%) and specificity (23.8%, 72.1–39.5%) were low based on detectable levels.

### Outcomes at follow-up: comparison of intervention and nonintervention groups

There was no significant difference in the adherence to AEDs based on self-reports (OR 1.00, 95% CI 0.71–1.40, p = 1.00), detectable (OR 1.46, 95% CI 0.74–2.90, p = 0.28), and optimal (OR 0.91, 95% CI 0.51–1.61, p = 0.74) drug levels, between the two groups (Table2). In addition, there was no statistically significant difference in mean blood concentrations of AEDs between the groups. PWE in the intervention group had higher scores than PWE in the nonintervention group for beliefs about cultural treatment (p = 0.001), lack of negative stereotypes (p = 0.001), and beliefs about causes of epilepsy (p = 0.04) (Table2). Seizure frequency was not different between the groups.

**Table 2:** Comparison of outcomes between the intervention and nonintervention groups at the end of the study

Variable	Intervention group (n = 303)		Nonintervention group (n = 278)		p-Value
	n	%	n	%	
Adherence					
Self-reported	193	63.7	177	63.7	1.00
Detectable level in blood	85/105	81.0	64/86	74.4	0.28
Optimal level in blood	50/105	47.6	43/86	50.0	0.74
AED levels in blood: mean level (SD)					
Phenobarbital	63	13.1 (11.9)	53	11.3 (10.8)	0.35
Phenytoin	46	2.1 (2.2)	41	2.4 (3.8)	0.70
Carbamazepine	34	3.3 (4.3)	48	3.7 (3.9)	0.66
Seizures					
Less frequent seizures	243	80.2	208	74.8	0.12
Seizure frequency (last 3 months)					
None	154	50.8	130	46.8	
1–3	89	29.4	78	28.1	
4–6	26	8.6	26	9.4	
>6	34	11.2	44	15.8	0.40

KEBAS	n	Mean (SD)	N	Mean (SD)	p-Value
Beliefs about causes of epilepsy	303	7.4 (2.6)	278	7.0 (2.7)	0.04
Beliefs about biomedical treatment	303	15.1 (15.1)	278	15.0 (1.9)	0.57
Beliefs about cultural treatment	303	12.8 (4.1)	278	11.6 (4.3)	<0.001
Beliefs about risks of epilepsy	303	7.3 (1.3)	278	7.4 (1.4)	0.27
Stereotypes about epilepsy	303	11.0 (3.8)	278	10.0 (3.9)	<0.001

If all the data were not available, both the numerator and denominators are provided. % column records percentage of those observed with the characteristic under consideration except for the items of KEBAS and levels of AEDs in blood where we have the mean (standard deviation) of the scores.

### Comparison of measures at baseline and follow-up

The proportion of PWE with therapeutic, detectable, or self-reported adherence to AEDs increased at follow-up from the baseline in both groups with larger increase recorded in the intervention group (Table3). In the intervention group, the adherence (measured by detectable levels of AEDs) improved from 36% to 81%, whereas the nonintervention group improved from 38% to 74%. Furthermore, there was a significant increase in proportion of PWE with less frequent seizures at follow-up compared with those at baseline in both groups; with larger increases in the intervention group. Overall there was a significant improvement in KEBAS scores for perceptions about PWE and reduction in traditional beliefs of treatment. There was improvement in KEBAS score for item on the perceptions of PWE at follow-up compared to baseline for the nonintervention group (10.0 at follow-up vs. 8.5 at baseline, p < 0.001). In the intervention group, there was a significant reduction in the beliefs about cultural treatment of epilepsy (12.8 at follow-up vs. 11.0 at baseline, p < 0.001) and negative perceptions of PWE (11.0 at follow-up vs. 8.4 at baseline, p < 0.001), but not in KEBAS scores for the causes, biomedical treatment, and risks of having epilepsy. For the nonintervention group, there was no observed difference in KEBAS scores for biomedical causes or treatment, traditional-treatment, and risks of epilepsy (Table3).

**Table 3:** Comparison of baseline and follow-up

Variable	Intervention group			Nonintervention group			Combined groups		
	Baseline	Follow-up	p-Value	Baseline	Follow-up	p-Value	Baseline	Follow-up	p-Value
Adherence (%)									
Self-reported	52/195 (26.7)	193/303 (63.7)	<0.001	54/199 (27.1)	177/278 (63.7)	<0.001	106/395 (26.9)	370/581 (63.7)	<0.001
Detectable blood levels of AED	71/195 (36.4)	85/105 (81.0)	<0.001	76/199 (38.2)	64/86 (74.4)	<0.001	147/394 (37.3)	149/191 (78.0)	<0.001
Optimal blood levels of AED	52/192 (27.1)	50/105 (47.6)	0.004	59/196 (30.1)	43/86 (50.0)	0.001	111/388 (28.6)	93/191 (48.7)	<0.001
Less frequent Seizures	187/303 (61.7)	243/303 (80.2)	<0.001	186/278 (66.9)	208/278 (74.8)	0.04	373/581 (64.2)	451/581 (77.6)	<0.001
KEBAS: mean (SD)									
Beliefs about causes of epilepsy	7.3 (2.8)	7.4 (2.6)	0.63	7.3 (2.9)	7.0 (2.7)	0.16	7.3 (2.8)	7.2 (2.6)	0.53
Beliefs about biomedical treatment	15.0 (2.0)	15.1 (1.9)	0.45	14.9 (2.2)	15.0 (1.9)	0.48	15.0 (2.1)	15.1 (1.9)	0.30
Beliefs about cultural treatment	11.0 (4.8)	12.8 (4.1)	<0.001	11.1 (4.9)	11.6 (4.3)	0.17	11.1 (4.8)	12.2 (4.2)	<0.001
Beliefs about risks of epilepsy	7.4 (1.3)	7.3 (1.3)	0.22	7.5 (1.2)	7.4 (1.4)	0.47	7.4 (1.2)	7.3 (1.3)	0.16
Stereotypes about epilepsy	8.4 (4.2)	11.0 (3.8)	<0.001	8.5 (4.6)	10.0 (3.9)	<0.001	8.5 (4.4)	10.5 (3.8)	<0.001

Items of KEBAS are reported as mean (standard deviation) of the scores. If all the data were not available, both the numerators and denominators are provided.

### Factors associated with improved adherence

Univariate analysis of factors associated with improved adherence, reduced seizures, and positive change in KEBAS are shown in Tables4, and Tables S1 and S2, respectively. From the multivariable analysis, reduced seizure frequency was associated with improved adherence based on optimal blood concentrations of AEDs (relative risk [RR] 1.72, 95% CI 1.19–2.47), but not with age (p = 0.11), sex (p = 0.08), and being injured (p = 0.36). Improved adherence was positively associated with positive change in beliefs about risks of epilepsy (RR 2.00, 95% CI 1.03–3.95) and having nontraditional religious beliefs (RR 2.01, 95% CI 1.01–3.99), but not with age, neurologic deficits, and stereotypes about epilepsy (Table5). Positive changes in KEBAS were associated with having tertiary education as compared to none (RR = 1.09, 1.05–1.14), but being injured (p = 0.088) and having less frequent seizures (p = 0.245) were not significantly associated with KEBAS.

**Table 4:** Univariate analysis for factors associated with improved therapeutic adherence adjusted for the intervention

Variable	Improved (n = 29)	No Improvement (n = 54)	RR (95% CI)	p-Value
Age: Mean (SD)	29.4 (17.8)	22.1 (13.6)	1.02 (1.00–1.03)	0.01
Sex				
Female	15 (51.7%)	24 (44.4%)	1	
Male	14 (48.3%)	30 (55.6%)	0.79 (0.44–1.43)	0.44
Injured				
No	17 (58.6%)	32 (59.3%)	1	
Yes	12 (41.4%)	22 (40.7%)	1.01 (0.56–1.84)	0.97
Educational level				
None	11 (37.9%)	21 (38.9%)	1	
Primary	16 (55.2%)	31 (57.4%)	0.97 (0.52–1.79)	0.91
Secondary	2 (6.9)	2 (3.7%)	1.60 (0.47–5.44)	0.46
Religion				
Traditional	7 (21.2%)	26 (44.0%)	1	
Nontraditional	22 (78.8%)	28 (56.0%)	2.10 (1.00–44.40)	0.05
Learning difficulties				
No	25 (86.2%)	43 (76.6%)	1	
Yes	4 (13.8%)	11 (20.4%)	0.71 (0.28–1.82)	0.48
Neurologic deficit				
No	24 (82.8%)	49 (90.7%)	1	
Yes	5 (17.2%)	5 (9.3%)	1.59 (0.81–3.11)	0.18
On polytherapy	6/18 (33.3%)	13/34 (38.2%)	0.82 (0.36–1.89)	0.65
Improved KEBAS: Mean (SD)				
Beliefs about causes of epilepsy	6 (20.7)	16 (29.6)	0.75 (0.35–1.61)	0.46
Beliefs about biomedical treatment	10 (34.5)	20 (37.0)	0.98 (0.53–1.83)	0.95
Beliefs about cultural treatment	8 (27.6)	7 (13.0)	1.02 (0.57–1.85)	0.94
Beliefs about risks of epilepsy	15 (51.7)	27 (50.0)	1.89 (1.04–3.45)	0.04
Stereotypes about epilepsy	22 (75.9)	30 (55.6)	1.86 (0.90–3.87)	0.10

Data are number of patients (%) except for items of KEBAS and age where we have the mean (standard deviation) of the scores. This analysis was done on 83 PWE who had nonoptimal levels of AEDs in the blood at baseline and had optimal levels of AEDs in blood at follow-up.

**Table 5:** Multivariable analysis for factors associated improved adherence based on therapeutic levels adjusted for the intervention

Variable	RR (95% CI)	p-Value
Age	2.10 (0.94–4.69)	0.07
Religion		
Traditional	1	
Nontraditional	2.01 (1.01–3.99)	0.05
Improved KEBAS		
Beliefs about risks of epilepsy	2.00 (1.01–3.95)	0.04
Stereotypes about epilepsy	1.98 (0.94–4.17)	0.07
Neurologic deficit		
No	1	
Yes	1.29 (0.51–3.26)	0.59

Nontraditional religious beliefs refer to Christianity and Islam.

## Discussion

This study tested the efficacy of a one-day educational intervention to improve adherence to AEDs in a resource-poor setting, where the treatment gap was moderately high (62%).^5^ We found no difference in adherence between the intervention and nonintervention groups after the intervention was implemented, but adherence was improved in both groups, and this improvement was associated with reduced seizure frequency. Compared with the nonintervention group, the intervention group was associated with improvements in knowledge about causes of epilepsy, fewer beliefs in negative stereotypes, and cultural/traditional treatment. Increase in knowledge about the risks associated with epilepsy and having nontraditional religious beliefs were important predictors of improved adherence in both groups combined.

### Adherence and education intervention

This study did not find any significant improvements in adherence between the intervention and nonintervention groups following the education intervention, although there was a >20% improvement in both groups combined as measured by AEDs in the blood. This lack of difference in adherence between the groups may have a number of explanations. First, the improvement in adherence may have been related to factors other than the intervention, for example, limited access and supply of AEDs. Secondly, the follow-up period may have been too short.^22^ Lastly, and most likely, the improved adherence in both groups could be explained by the sharing of knowledge between the groups.^23^ Such an occurrence may be prevented by using a cluster-randomized design.^24^ Contamination could be caused by health workers and traditional healers while they attended to the patients in the nonintervention group. This could also have underestimated the difference in KEBAS between the two groups.

### Comparison of adherence between baseline and follow-up

Despite the lack of significant improvement in adherence between the intervention and nonintervention groups, there was an overall significant absolute improvement (>34%) in adherence at follow-up compared with baseline in both groups, suggesting that factors other than the intervention may have caused the improvement in adherence that was observed. These factors may also explain significant reduction in seizure frequency with increased utilization of AEDs and higher levels for AEDs. In other studies, reduction in seizure frequency is associated with improved adherence to AEDs, which may be related to multiple factors.^8^,^25^ Many other studies have explored usefulness of interventions by comparing baseline and follow-up measures only,^26^–^28^ although the results would be more reliable with a comparison group in a randomized controlled trial design.^29^ However, these improvements in both groups should be interpreted cautiously as they may be related to other factors, for example, sensitization of the community and the placebo effect.

### Beliefs about epilepsy and adherence

There was improvement in KEBAS, but this may not have directly resulted in improved adherence. Even though negative beliefs about epilepsy influence adherence for PWE,^2^,^5^ this study demonstrates that the resulting improvement may not always translate into improvement in adherence. This inconsistency may be explained in two ways. First, a longer period of follow-up may be needed for an effect on adherence to be seen. This Hawthorne effect may remain even after the conclusion of a study resulting in concomitant change in adherence.^30^ The change in beliefs about epilepsy in this study is in a direction associated with improved adherence in other studies.^31^,^32^ Secondly, other different interventions such as reminders about prescriptions^33^ and improving access to AEDs may be required to change adherence. There is need to apply further interventions, since ETG is still high in the area, although it was lowered from 70.3% in 2003 to 62.4% in 2010.^5^,^34^

### Factors associated with changes in the outcomes

Improved therapeutic adherence was positively correlated with being a nontraditionalist and positive changes in risks of epilepsy, possibly because these patients were more likely to believe in and seek biomedical treatment. Improvement in KEBAS was associated with being educated, perhaps because of the improved level of understanding in these people. These conclusions show that improvement in adherence depends on several factors but not only on education.

### Strengths and limitations

This is the first trial to test the usefulness of an educational intervention to reduce the ETG in a resource-poor setting. The groups were selected randomly, ensuring similarities between the two groups to avoid any bias in the outcome. The outcome was measured with validated tools that were developed for use in this setting. The limitations of this study are the following: the intervention was advanced only once, inability to prevent spread of knowledge by health care workers, traditional healers and participants in the intervention group to those in the non-intervention group, the duration of follow-up was relatively short and therefore the full effect of the education intervention may not have been apparent. In addition, the majority of the study participants did not give blood.

## Conclusion

Health education improves knowledge about epilepsy, but once only contact does not improve adherence. However, sustained education may in part improve adherence in future studies. The findings of this study are useful in planning interventions aimed at reducing the treatment gap in other similar settings in Africa and across the world.

## Supporting Information

Figure S1 This diagram represents the design of the study.

Table S1 Univariate analysis for factors associated with reduction in seizures adjusted to the intervention. **Table S2** Univariate analysis for factors associated with positive changes in KEBAS adjusted for the intervention.
