# Supplementary figures and images for: Evaluation of Kilifi Epilepsy Education Programme: A randomized controlled trial
Source: Epilepsia. 2014 Jan 21;55(2):344–52. doi: 10.1111/epi.12498 (PMC4233970; doi:10.1111/epi.12498)

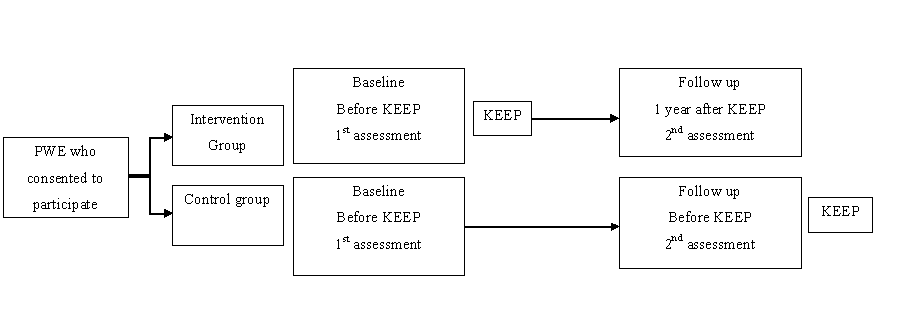

Supplement: Figure S1 — This diagram represents the design of the study. [file epi0055-0344-SD1.tif]
