# Supplementary material for: Evaluation of Kilifi Epilepsy Education Programme: A randomized controlled trial
Source: Epilepsia. 2014 Jan 21;55(2):344–52. doi: 10.1111/epi.12498 (PMC4233970; doi:10.1111/epi.12498)
Supplement: Table S1 — Univariate analysis for factors associated with reduction in seizures adjusted to the intervention. Table S2 Univariate analysis for factors associated with positive changes in KEBAS adjusted for the intervention. [file epi0055-0344-SD2.docx]

**Supplementary table 1: univariate analysis for factors associated with reduction in seizures adjusted to the intervention.**

| **Variable** | **Reduction seizures**  (n=244) | **No reduction in seizures**  (n=103) | **RR(95%CI)** | **P value** |
| --- | --- | --- | --- | --- |
| **Age** : Mean(SD) | 21.9 (15.3) | 25.7 (16.5) | 1.00(0.99-1.00) | 0.08 |
| **Sex** |  |  |  |  |
| Female | 112 (45.9%) | 58 (56.3%) | 1 |  |
| Male | 132 (54.1%) | 45 (43.7%) | 1.13(0.98-1.30) | 0.08 |
| **Injured** |  |  |  |  |
| No | 154 (63.1%) | 49 (47.6%) | 1 |  |
| Yes | 90 (36.9%) | 54 (52.4%) | 0.83(0.72-0.97) | 0.02 |
| **Religion** |  |  |  |  |
| Traditional | 96/244 (39.3%) | 45/103 (47.7%) | 1 |  |
| Non-traditional | 148/244 (60.7%) | 58/103 (56.3%) | 1.04(0.90-1.20) | 0.57 |
| **Educational level** |  |  |  |  |
| None | 108(44.3%) | 51(49.5%) | 1 |  |
| Primary | 118(48.4%) | 46(44.7%) | 1.06(0.92-1.22) | 0.42 |
| Secondary | 15(6.2%) | 5(4.9%) | 1.10(0.83-1.46) | 0.50 |
| Tertiary | 3(1.2%) | 1(1.0%) | 1.11(0.60-2.05) | 0.74 |
| **Learning difficulties** |  |  |  |  |
| No | 165/226(73.0%) | 65/96(67.7%) | 1 |  |
| Yes | 61/226(27.0%) | 31/96(32.3%) | 0.93(0.79-1.10) | 0.40 |
| **Neurological deficit** |  |  |  |  |
| No | 193/226(85.4%) | 77/96(80.2%) | 1 |  |
| Yes | 33/226((14.6%) | 19/96(19.8%) | 0.88(0.71-0.10) | 0.26 |
| **On polytherapy** | 46/131(35.1%) | 29/71(40.9%) | 0.91(0.74-1.13) | 0.39 |
| **Improved therapeutic adherence** | 19/37(51.4%) | 2/19(10.5%) | 1.82(1.15-2.88) | 0.01 |

Data are number of patients (%) except for age where we have the mean (standard deviation). If all the data were not available, both the numerator and denominators are provided.

**Supplementary table 2: univariate analysis for factors associated with positive changes in KEBAS adjusted for the intervention.**

| **Variable** | **Improved**  (n=534) | **No Improvement**  (n=47) | **RR(95%CI)** | **P value** |
| --- | --- | --- | --- | --- |
| **Age** : Mean(SD) | 22.2(15.7) | 23.3(15.3) | 1.00(0.99-1.00) | 0.64 |
| **Gender** |  |  |  |  |
| Female | 259(48.5%) | 22(46.8%) | 1 |  |
| Male | 275(51.5%) | 25(53.2%) | 0.99(0.95-1.04) | 0.82 |
| **Injured** |  |  |  |  |
| No | 346(64.8%) | 23(48.9%) | 1 |  |
| Yes | 188(35.2%) | 24(51.1%) | 0.95(0.89-1.00) | 0.05 |
| **Educational level** |  |  |  |  |
| None | 245(45.9%) | 23(48.9%) | 1 |  |
| Primary | 251(47.0%) | 23(48.9%) | 1.00(0.95-1.05) | 0.93 |
| Secondary | 31(5.8%) | 1(2.13%) | 1.06(0.99-1.14) | 0.10 |
| Tertiary | 7(1.3%) | 0 | 1.09(1.05-1.14) | <0.001 |
| **Learning difficulties** |  |  |  |  |
| No | 370/491(75.4) | 30/44(68.2) | 1 |  |
| Yes | 121/491(24.6) | 14/44(31.8) | 0.97(0.91-1.03) | 0.34 |
| **Neurological deficit** |  |  |  |  |
| No | 422/491(86.0%) | 35/44(80.0) | 1 |  |
| Yes | 69/491(14.1%) | 9/44(20.5) | 0.96(0.88-1.04) | 0.32 |
| **On polytherapy** | 98/279(35.1%) | 13/29(44.8%) | 0.96(0.89-1.04) | 0.32 |
| **Religion** |  |  |  |  |
| Traditional | 221(41.4%) | 18(38.3%) | 1 |  |
| Non-traditional | 313(58.6%) | 29(61.7%) | 0.99(0.94-1.04) | 0.66 |
| **Less frequent seizures** | 348/534(65.2%) | 25/47(53.2%) | 1.04(0.99-1.10) | 0.12 |

Data are number of patients (%) except for age and years with seizures where we have the mean (standard deviation). If all the data were not available, both the numerator and denominators are provided. Non-traditional religious beliefs refer to Christianity and Islam. Improved KEBAS is defined as an improvement in scores in at least one of the five items of KEBAS at follow-up compared to baseline.
